# Lactobionic acid production by glucose–fructose oxidoreductase from *Zymomonas mobilis* expressed in *Escherichia coli*

**DOI:** 10.1007/s10529-015-1887-0

**Published:** 2015-06-20

**Authors:** Kamila Goderska, Wojciech Juzwa, Artur Szwengiel, Zbigniew Czarnecki

**Affiliations:** Poznan University of Life Sciences, Faculty of Food Science and Nutrition, Department of Fermentation and Biosynthesis, Institute of Food Technology of Plant Origin, Wojska Polskiego 31, 60-624 Poznan, Poland; Poznan University of Life Sciences, Faculty of Food Science and Nutrition, Department of Biotechnology and Food Microbiology, Wojska Polskiego 48, 60-627 Poznan, Poland

**Keywords:** *Escherichia coli*, Glucose–fructose oxidoreductase, Lactobionic acid, Lactose, Whey, *Zymomonas mobilis*

## Abstract

**Objectives:**

To use the glucose–fructose oxidoreductase (GFOR) from *Zymomonas mobilis* and expressed in *Escherichia coli* for lactobionic acid production by conversion of lactose from whey.

**Results:**

The highest concentrations of lactobionic acid (3.2 mg ml^−1^) during oxidation of whey-derived lactose by *E. coli* was at 24 h. Introduction of GFOR gene from *Z. mobilis,* into *E. coli* improved enzyme yields compared to what is obtainable by fermentation of the donor strain. The production of lactobionic acid by *E. coli* was 2.6-times higher than by *Z. mobilis.*

**Conclusions:**

Recombinants of *E. coli* overexpressing the GFOR gene from *Z. mobilis* produced higher amount of lactobionoic acid from whey-derived lactose.

## Introduction

**Fig. 1:**
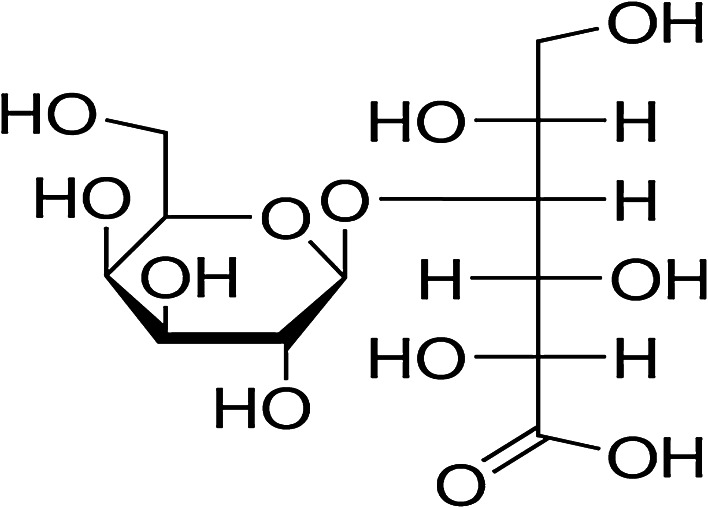
Lactobionic acid

Lactobionic acid (LBA) (Fig. 1) is a relatively new product derived from lactose oxidation. It has potential applications as a bioactive compound. It is an aldonic acid obtained from the oxidation of lactose, with application as an ingredient in foods and pharmaceutical products, because of its antioxidant, chelating and humectant properties (Gutierrez et al. 2012). The chemical structure of LBA comparises a galactose moiety linked to a gluconic acid molecule via an ether-like linkage. LBA is also used in calcium supplementation and represents a new ingredient in skin-care products with potent antioxidant and humectant properties. In the food industry, LBA is used as acidulant with a sweet taste, as filler in cheese production, as firming agent, and to fortify functional drinks with essential minerals such as Fe and Cu.

Glucose–fructose oxidoreductase and gluconolactonase (GFOR/GL) oxidizes not only glucose but also seven other aldose sugars to produce the corresponding organic acids; in particular, lactose is oxidized to LBA (Pedruzzi et al. 2007). Druliolle et al. (1997) have shown that the electrocatalytic oxidation of lactose on noble metal electrodes in alkaline medium permits yields LBA with a high selectivity.

Lactose is mainly used as an ingredient in foods, beverages and confectionery products. It is also extensively employed as diluent in tablets and carrier of medicines in the pharmaceutical industry. Nevertheless, the use of lactose is limited in many applications, because of its low sweetness and solubility, as well as due to the intolerance of some population segments, and only a small amount of lactose is employed as a raw material for producing fine chemicals (Gutierrez et al. 2011).

However, the worldwide surplus and low cost of lactose have motivated research on innovative processes for producing valuable lactose derivatives, and expanding their applications in the food, pharmaceutical and chemical industries. Significant developments include the production of highly valued pharmaceutical products and functional food ingredients, such as lactitol, LBA, lactosucrose, lactulose and galacto-oligosaccharides, some of which have become commercially successful (Gänzle et al. 2008).

A new carbohydrate oxidase, lactose oxidase, with high specificity of oxidizing the disaccharide lactose to LBA was found by Ahmad et al. (2004). In food technology, LBA may find applications due to its ability to form mineral salt complexes and its presumed prebiotic effect. One of the new applications is the conversion of lactose in milk to LBA and exploiting the desirable characteristics of LBA to replace proteins and/or fats in process, cheeses and cream cheese (Ahmad et al. 2004).

## Materials and methods

### Microorganisms and cultivation

*Zymomonas mobilis* subsp. *mobilis* CCM 2770 and CCM 3883 (obtained from Czech Collection of Microorganisms, Brno, Czech Republic) was used. It was grown in a 300 ml flask containing 50 ml modified medium (20 g glucose l^−1^, 10 g yeast extract l^−1^, 1 g KH_2_PO_4_ l^−1^, 1 g (NH_4_)_2_SO_4_ l^−1^, and 0.5 g MgSO_4_·7H_2_O l^−1^) and then incubated at 30 °C for 24 h. The subculture was then inoculated into a 5 l fermentors (Biostat Braun Biotech International, Germany) containing 4 l medium, with aeration at 1 vvm and agitation at 120 rpm at 30 °C. After cultivation for 96 h, cells were harvested by filtration. *Escherichia coli* XL1-Blue MRF’ [Δ(mcrA)183, Δ(mcrCB-hsdSMR-mrr)173, endA1 supE44, thi-1, recA1, gyrA96, relA1, lac [F’ proAB lacI^q^ZΔM15 Tn10 (Tet^r^)]] (Stratagene) was inoculated into a 300 ml flask containing 50 ml LB medium (10 g tryptone l^−1^, 5 g yeast extract l^−1^, and 10 g NaCl l^−1^) medium and then incubated at 30 °C for 24 h with agitation at 120 rpm. The isolation of genomic DNA was preceded by 24 h cultivation of *Z. mobilis* cells on modified medium.

### Genomic DNA isolation from *Z. mobilis*

*Z. mobilis* cells were suspended in 200 μl lysis buffer I (RNase A, 100 μg/ml; 50 mM Tris/HCl,10 mM EDTA, pH 7.5) and transferred into Eppendorf tubes containing approx. 50 μl glass beads (115–200 μm diam.). Cells were disintegrated at 30 rotations/min for 5 min and centrifuged at 15,000×*g* for 5 min. Supernatants were transferred to the fresh Eppendorf tubes containing 200 μl of buffer II (0.2 M NaOH, 1 % SDS) and held on ice for 30 min. 200 μl phenol/CIAA (CIAA—chloroform + isoamylic alcohol) mixture, was added, vortexed and centrifugation at 10,000×*g* for 4 min at RT. The aqueous phase was aspirated and plasmid DNA was precipitated using 450 μl 2-propanol and centrifugation at 15,000×*g* for 5 min. After washing of DNA pellet using 100 μl of 75 % (v/v) ethanol, genomic DNA was resuspended in 50 μl of TE buffer.

### Cloning of glucose–fructose oxidoreductase (GFOR) genomic sequence into pQE-30 vector (Qiagen)

Genomic DNA was isolated using the automated system for DNA and protein isolation and purification Maxwell 16 (Promega) and the alkaline lysis method. Genomic DNA was isolated from 2770 and 3883 *Z. mobilis* strains.

Amplification of the GFOR gene was conducted using modified primers: Forward (F)—GFORzmSac_F1 and Reverse (R)—GFORzm*Hin*d_R. The endonuclease *Sac*I and *Hin*dIII recognition sites were placed in the forward and reverse primers, respectively. A sequence recognized by *Sac*I (5′-ATGAGCTCCTGGTTCCGCGTGGATCTGATTACAGCAACTTCGACAAGA-3′) was placed in the F primer and a sequence recognised by the *Hin*dIII (5′-ATAAAGCTTTTAACCCAAATAGGTTAAGTCAGA-3′) was placed in the R primer. Additionally a sequence recognized by the thrombine enzyme was placed in the F primer. PCR of 25 μl was conducted as follows: 94 °C, 60 s; 58 °C, 50 s; 72 °C, 150 s; 30 cycles. The reaction mixture contained 100 ng *Z. mobilis* genomic DNA, 20 pM primers, 7.5 nM dNTP, buffer and 1 U DNA *Taq* polymerase (Qiagen). PCR product was evaluated by 1.5 % agarose gel electrophoresis, extracted with liquid nitrogen and cleaned with the use of phenol/chloroform method. PCR product was then digested by *Sac*I and *Hin*dIII, purified and ligated with the pQE-30 vector, which was digested with the same endonucleases.

### Preparative scale batch reactions

Batch cultivations were performed in a 2 l bioreactor (Biostat Braun Biotech International, Germany) with 1 l of whey (2 % w/v lactose) at 30 °C. An inoculum of 10 % (v/v) was used. An efficient two-stage pH-shifted bioconversion strategy was adopted as previously described (Alonso et al. 2011): pH was controlled above 6.5 (pH was left uncontrolled above this value during the growth phase and subsequently maintained at 6.5) by means of computer-controlled peristaltic pumps via automatic addition of 2 M NaOH. These prior conditions were applied to all cultivations unless otherwise specified. Cultivations were carried out in duplicate as independent experiments.

### Rennet whey preparation

Cheese rennet whey (Poznan, Poland) was diluted with distilled water (1:1 v/v) and adjusted to pH 6.5 with 6 M NaOH prior to sterilization using a tangential microfiltration device equipped with a PVDF membrane-cassette of 0.22 μm pore size.

### Disruption of cells

Washed bacteria in acetic buffer (0.05 M, pH 5.0) were disrupted by ultrasonication for 30 min. Cell was removed by centrifugation at 15,000×*g* for 30 min at 4 °C. To obtain the high speed supernatant fraction, cell free extracts were centrifuged at 15,000×*g* for 30 min. This fraction was used either immediately or stored at −20 °C.

### Estimation of protein

The protein content of cell-free extracts and high speed supernatants were determined by using the method of Bradford with bovine serum albumin as standard.

### Analysis of lactose and lactobionic acid

#### LC–ESI–MS analysis

Hydrophilic interaction chromatography (HILIC) with electrospray ionization mass spectrometry (HILIC–ESI–MS) analysis was performed using a Dionex UltiMate 3000 UHPLC (Thermo Fisher Scientific, Sunnyvale, CA, USA) coupled to a Bruker maXis impact ultrahigh resolution orthogonal quadrupole-time-of-flight accelerator equipped with an ESI source and operated in positive-ion mode (Bruker Daltonik, Bremen, Germany). The chromatographic separation was achieved with a ACQUITY UPLC BEH Amide 1.7 µm column, 150 × 2.1 mm (Waters). The ESI–MS settings were as follows: capillary voltage 4500 V, nebulizing gas 1.8 bar, and dry gas 9 l min^−1^ at 200 C. The scan range was from mass-to-charge ratio (m/z) 80–1300. The mobile phase was acetonitrile/water (75:25 v/v) containing 0.1 % NH_4_OH at 0.2 ml min^−1^ with a isocratic elution. The sample injection was 3 µl. The column was set to 40 °C. The ESI–MS system was calibrated using sodium formate clusters introduced by loop-injection at the beginning of the LC–MS run. The LC–MS data were processed using Data Analysis 4.1 software (Bruker Daltonik, Bremen, Germany). Molecular ions: [M+H]^+^, [M+NH_4_]^+^, [M+Na]^+^ for LBA (359.118, 376.145, 381 100 m/z); [M+H]^+^, [M+Na]^+^ for lactose (343.124, 365.105 m/z) were extracted from full scan chromatograms and peak areas were integrated. Ribose ([M+Na]^+^) with retention time (t_R_) 4 min and saccharose at 7.5 min ([M+NH_4_]^+^, [M+Na]^+^) were used as an internal standards for LBA (2 min) and lactose (9.3 min), respectively. Internal standards were added in a constant amount to samples, the blank and calibration standards (ribose at 10 µg ml^−1^ and saccharose at 20 µg ml^−1^). The extraction window of individual ion chromatograms was ±0.01 m/z units. The compounds present in each sample were identified by comparing their retention times with those of standards, and based on molecular mass and structural information from the MS detector.

**Fig. 2:**
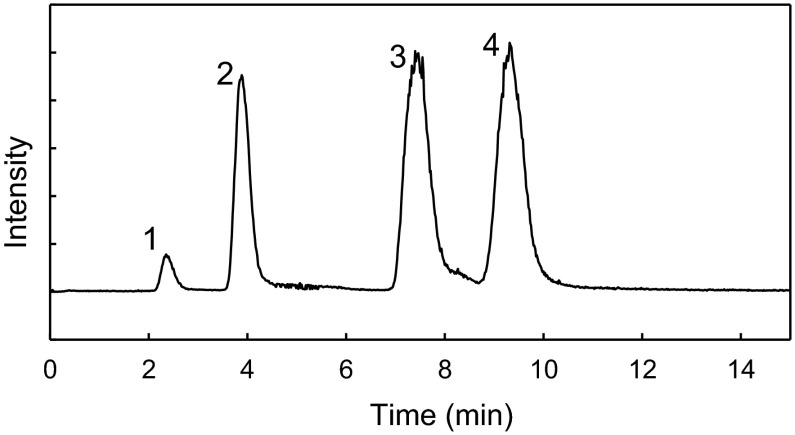
The extracted ion chromatogram (EIC) of LC–MS separation of sample (diluted 1000 times) with internal standards (ISTD); *1* lactobionic acid (LA) = 3 µg ml^−1^, *2* ribose (ISTD for LA) = 10 µg ml^−1^, *3* saccharose (ISTD for L) = 20 µg ml^−1^, *4* lactose (L) = 11 µg ml^−1^

A water solution of standards was used for calibration by plotting the ratio of the analyte signal to the internal standard signal (Relative response—y) as a function of the analyte concentration of the standards to the concentration of internal standard (Relative concentration—x). The concentration of LBA and lactose were calculated according to formulas—lactobionic acid: y = 0.524357x−0.00876, R^2^ = 0.9999, lactose: y = 1.428183x−0.027, R^2^ = 0.9998. The limit of detection (LOD) was calculated as the resulting concentration after using a signal-to-noise criterion of 3. The LOD for analytes was <0.1 µg ml^−1^. The lower limit for quantification (LLQ) was determined as the lowest concentration for which the obtained relative standard deviation was smaller than 10 %. The LLQ for analytes was about ~0.5 µg ml^−1^. The linear range was determined as two points between LLQ and the highest concentration of a compound, which still maintains a good linearity (with a threshold R^2^ value equal to or above 0.99). The estimated linear range for LBA and lactose was in range 0.5–25 and 0.5–15 µg ml^−1^ respectively (Fig. 2).

## Results

Genomic DNA isolation from *Z. mobilis* and quantitative and qualitative analysis of *Z. mobilis* genomic DNA isolates.

The prepared gene construct contained genomic sequence coding for GFOR from *Z. mobilis.* The concentration of genomic DNA isolated from *Z. mobilis* strains was evaluated with a NanoDrop spectrophotometer. Qualitative analysis was performed using 1.2 % agarose gel electrophoresis to check the degradation status of DNA. The concentrations of *Z. mobilis* genomic DNA in isolated samples were: *Z. mobilis* 2770 isolated with the alkaline lysis method—125 ng μl^−1^; *Z. mobilis* 3883 isolated with the alkaline lysis method—198 ng μl^−1^; *Z. mobilis* 2770 isolated with the automatic method—188 ng μl^−1^; *Z. mobilis* 3883 isolated with the automatic method—179 ng μl^−1^.

**Fig. 3:**
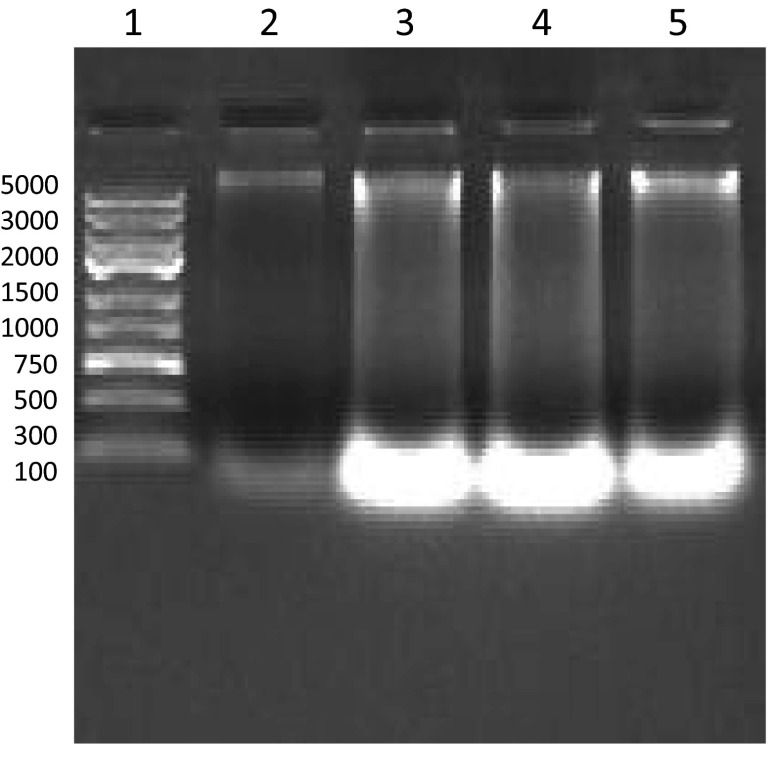
Qualitative analysis of genomic DNA samples isolated from *Zymomonas mobilis* strains with the 1.2 % agarose gel electrophoresis. Lanes 2–3, genomic DNA isolated with the alkaline lysis method from *Zymomonas mobilis* 2770 strain and 3883 strain respectively; lanes 4–5, genomic DNA isolated with the automatic method from *Zymomonas mobilis* 2770 strain and 3883 strain respectively; lane 1, size marker GeneRuler Express DNA Ladder (Fermentas)—band sizes are as follow: 100, 300, 500, 750, 1000, 1500, 2000, 3000 and 5000 bp

Figure 3 demonstrates the qualitative analysis of genomic DNA samples isolated from *Z. mobilis* strains with the 1.2 % agarose gel electrophoresis. Lanes 2–3, genomic DNA isolated with the alkaline lysis method from *Z. mobilis* 2770 and 3883, respectively; lanes 4–5, genomic DNA isolated with the automatic method from *Z. mobilis* 2770 and 3883, respectively; lane 1, size marker GeneRuler Express DNA Ladder (Fermentas). The quality of high molecular weight genomic DNA as represented by bands over the higher size marker band (5000 bp) was satisfactory with no significant signs of DNA degradation. The visible bands below the lowest size marker band (100 bp) may be represented by low molecular weights RNA molecules. The used methods of genomic DNA isolation occurred susceptible for the high RNA contents.

### Preparing recombinants of *E. coli* cells overexpressing the GFOR gene from *Zymomonas mobilis*

Amplification of GFOR gene was conducted using primers enabling cloning into pQE-30 vector and purification of recombinant glucose–fructose oxidoreductase protein. Prior to appropriate PCR amplification, an optimization step was involved with the use of the gradient of annealing temperatures (54, 56, 58, 60, 62 and 64 °C). This step was aimed at optimizing the amplification of GFOR gene specific sequence. The optimal annealing temperature was selected based on no visible un-specific amplicons on agarose gel electropherogram. PCR product was evaluated by 1.5 % agarose gel electrophoresis, extracted with liquid N_2_ and cleaned using phenol/chloroform. The PCR product was digested by *Sac*I and *Hin*dIII, purified and ligated with the pQE-30 vector, which was digested with the same endonucleases. The pQE-30 vector comprise together with ATG a sequence coding for 6-histidine residues facilitating the isolating and cleaning procedure of overexpressed glucose–fructose oxidoreductase protein. The products of ligation were used for transformation of competent *E. coli* strain XL1 Blue. Recombinants were examined for the presence of insert by the digestion of isolated plasmid DNA with endonucleases used for cloning. The inserts were sequenced using automated genetic analysers (Applied Biosystems Prism). The sequencing results revealed 99 % of sequence identity with no gaps comparing to the subject *Zymonomas mobilis* glucose–fructose oxidoreductase (GFOR) gene according to BLAST search.

**Fig. 4:**
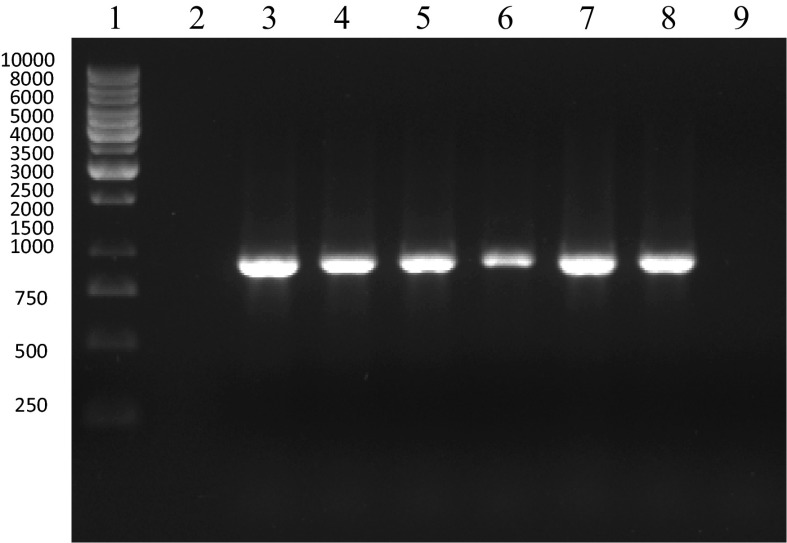
Electrophoretic analysis of the amplification of the sequence coding for GFOR gene (912 bp) with the use of PCR with the forward and reverse primers containing sequences recognized by the *Sac*I and *Hin*dIII enzymes respectively for cloning into vector pQE-30 (Qiagen). Lanes 3–8, amplicons of GFOR gene; lanes 2 and 9, negative control (no template); lane 1, size marker GeneRuler 1 kb DNA Ladder (Fermentas)—band sizes: 250, 500, 750, 1000, 1500, 2000, 2500, 3000, 3500, 4000, 5000, 6000, 8000 and 10,000 bp

Results of the amplification of the sequence coding for GFOR gene (912 bp) with the use of PCR with the forward and reverse primers containing sequences recognized by the *Sac*I and *Hin*dIII enzymes respectively for cloning into vector pQE-30 (Qiagen) are shown in Fig. 4.

**Fig. 5:**
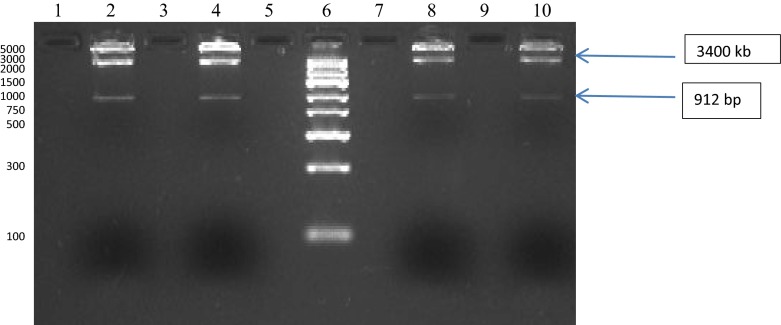
Analysis of selected recombinants after digestion of plazmid DNA with *Sac*I and *Hin*dIII restriction enzymes for the presence of cloned sequence coding for *Z. mobilis* GFOR gene into pQE-30 vector. Electrophoresis was conducted in 1.5 % agarose gel. Lanes 2, 4, 8 and 10, products of hydrolysis of recombinants: GF 11, GF 13, GF14, GF 15; lane 6, size marker GeneRuler Express DNA Ladder (Fermentas) – band sizes are as follow: 100, 300, 500, 750, 1000, 1500, 2000, 3000 and 5000 bp

Restriction analysis was performed for the selection of recombinants obtained after ligation with pQE-30 vector. Figure 5 demonstrates the analysis of selected recombinants after digestion of plasmid DNA with *Sac*I and *Hin*dIII restriction enzymes for the presence of cloned sequence coding for *Z. mobilis* GFOR gene into pQE-30 vector. Electrophoresis was conducted in 1.5 % agarose gel. Lanes 1–4, products of hydrolysis of recombinants: GF 11, GF 13, GF14, GF 15; lane 5, size marker GeneRuler Express DNA Ladder.

Recombinants GF 11, GF 13, GF14, GF 15 were selected after sequencing for overexpression experiments in order to evaluate GFOR activity. The whole plasmids isolated from recombinants were used as templates for sequencing of the GFOR gene using the insert specific primers: F (5′-ACCGATTACAGCAACTTCGA-3′) and R (5′- ACCCAAATAGGTTAAGTCAGA-3′) flanking the insert 5′- and 3′-ends.

Table 1 presents changes in the contents of lactose and LBA during oxidation of whey-derived lactose by enzymes produced by microorganisms *Z. mobilis* and *Escherichia coli*. The highest concentrations of LBA during oxidation of whey-derived lactose by *Escherichia coli* was after 24 h and 30 °C. Introduction of the glucose–fructose oxidoreductase gene into the recipient strain resulted in a *E. coli* production strain with significantly improved enzyme yields (50 %) compared to what is obtainable by fermentation of the donor strains of *Z. mobilis* 2770 and 3883.

**Table 1:** Changes in contents of lactose and lactobionic acid during oxidation of whey-derived lactose by Zymomonas mobilis and* Escherichia coli* bacteria

Strain of bacteria	Time (h)	Lactose (mg ml^−1^)			Lactobionic acid (mg ml^−1^)		
*E.coli* F_5_16 (recombinant F11)	24	3.21	3.03	3.12	5.37	1.05	3.21
*E.coli* F_5_24 (recombinant F15)	24	2.41	2.53	2.47	5.71	0.65	3.18
*Z.mobilis* 2770	24	2.59	2.77	2.68	0	0	0
*Z.mobilis* 3881	24	2.28	3.06	2.67	0.77	0.67	0.72
	48	2.26	2.47	3.04	0.75	0.73	0.74
	72	2.20	2.34	2.27	0.75	0.66	0.71
*Z.mobilis* 3882	24	2.77	2.40	2.59	0	0	0
	48	2.58	2.34	2.46	1.18	1.28	1.23
	72	2.39	2.74	2.56	1.22	1.22	1.22
*Z.mobilis* 3883	24	2.84	2.72	2.78	0	0	0
	48	2.23	2.53	2.38	0.74	0.99	0.87
	72	2.23	2.81	2.52	0.74	0.88	0.81
*Z.mobilis* 3884	24	2.51	2.68	2.6	0	0	0

The starting concentration of lactose was 10 mg/ml (3 repeats of the experiment)

## Discussion

Ahmad et al. (2004) reported toxicological investigations undertaken to evaluate the safety of a liquid enzyme concentrate, lactose oxidase, from *Microdochium nivale*. The enzyme was expressed in *Fusarium venenatum*, was produced in submerged fermentation and was recovered by purification/concentration from the culture broth. This significantly improved enzyme yields compared to what had been obtained by fermentation of the donor strain.

The enzyme (GFOR) was expressed by a strain of *E. coli* and was produced by submerged fermentation and recovered by purification/concentration of the culture broth. In our study, introduction of the GFOR gene into the recipient strain resulted in a *E. coli* production strain with significantly improved enzyme yields compared to what is obtainable by fermentation of the donor strain. The studies have compared production of LBA by *E. coli* and *Z. mobilis*.

Strict legal regulations concerning environmental protection oblige food producers to implement rational waste management instead of wastes being discharged to natural water bodies. For this reason whey is now being increasingly used in food technology.

Lactobionic acid as a product of lactose oxidation is mainly obtained on a commercial scale using microbiological methods and by catalytic oxidation in which the aldehyde group at carbon C1 is oxidized to the carbonyl group. Microbiological production of LBA consists in the enzymatic oxidation of lactose using mainly *Pseudomonas* spp*., Pseudomonas taetrolens, Acetobacter nivale* and *Z. mobilis.*

The highest concentrations of LBA (average 3.2 mg ml^−1^) during oxidation of whey-derived lactose by *E. coli* were recorded after 24 h. Introduction of the GFOR gene into *E. coli* resulted in production strain with significantly improved enzyme yields (about 50 %) compared to what was obtained by fermentation of *Z. mobilis* 2770 and 3883. Optimal conversions were at 38 °C and pH 6.4. It was also essential to maintain an appropriate concentration of O_2_ at about 14 %. On the basis of an experiment using *M. nivale* was shown.

## Conclusions

Introduction of the GFOR gene from *Z. mobilis*, into *E. coli* improved enzyme yields compared to what is obtainable by fermentation of the donor strain. The production of LBA by *Escherichia coli* was 2.6 times higher than by *Z. mobilis*.
